# Novel Ultrasound-Guided Popliteal Fascial Retinaculum Injection for Symptomatic Knee Osteoarthritis: A Technical Report With Cadaveric Dye-Distribution Study and Step-by-Step Procedural Description

**DOI:** 10.7759/cureus.107760

**Published:** 2026-04-26

**Authors:** Sang-Hyun Kim, U-Young Lee, Yonghyun Yoon, Teinny Suryadi, Daniel Chiung-Jui Su, Wei-Chuan Wang, King Hei Stanley Lam

**Affiliations:** 1 Anatomy, College of Korean Medicine, Woosuk University, Jeonbuk-do, KOR; 2 Anatomy, Catholic Institute for Applied Anatomy, College of Medicine, Catholic University of Korea, Seoul, KOR; 3 Orthopaedics, International Academy of Musculoskeletal Medicine, Hong Kong, HKG; 4 Orthopaedics, International Academy of Regenerative Medicine, Incheon, KOR; 5 Orthopaedics, MSKUS, San Diego, USA; 6 Orthopaedic Surgery, Hallym University Kangnam Sacred Heart Hospital, Seoul, KOR; 7 Orthopaedic Surgery, Incheon Terminal Orthopedic Surgery Clinic, Incheon, KOR; 8 Physical Medicine and Rehabilitation, Medistra Hospital, Jakarta Pusat, IDN; 9 Physical Medicine and Rehabilitation, Synergy Clinic, Jakarta, IDN; 10 Physical Medicine and Rehabilitation, Hermina Podomoro Hospital, Jakarta, IDN; 11 Physical Medicine and Rehabilitation, Chi Mei Medical Center, Tainan, TWN; 12 Physical Medicine and Rehabilitation, Ultra Clinic, Taipei City, TWN; 13 Faculty of Medicine, The Chinese University of Hong Kong, New Territories, HKG; 14 Faculty of Medicine, The University of Hong Kong, Hong Kong, HKG; 15 The Board of Clinical Research, The Hong Kong Institute of Musculoskeletal Medicine, Kowloon, HKG; 16 The Board of Clinical Research, The International Association of Musculoskeletal Medicine, Kowloon, HKG; 17 Research, International Academy of Regenerative Medicine, Incheon, KOR

**Keywords:** 5% dextrose, cadaveric dye-distribution study, hydrodissection, knee osteoarthritis, popliteal fascia, popliteus, posteromedial knee, semimembranosus, technical report, ultrasound-guided injection

## Abstract

Knee osteoarthritis (OA) is increasingly understood as a whole-joint disorder involving not only articular cartilage but also synovium, capsule, ligaments, tendon insertions, and adjacent periarticular soft tissues. Fascial and interfascial tissues have attracted growing interest in musculoskeletal medicine because of their continuity, force-transmission properties, sensory innervation, and potential relevance to pain generation and movement dysfunction.

We describe a novel ultrasound-guided posteromedial knee injection targeting the posteromedial fascial-periarticular convergence zone, referred to operationally in this report as the “popliteal fascial retinaculum.” This term is used as a descriptive procedural framework rather than a formally established anatomical structure. This convergence region involves the semimembranosus insertion, popliteus, oblique popliteal ligament (OPL), posterior capsule, proximal superficial medial collateral ligament (MCL), and adjacent popliteal fascial layers. The technique uses a structured scanning sequence beginning with transverse safety mapping of the popliteal neurovascular bundle, followed by posteromedial landmark identification and longitudinal localization of the target convergence zone. A 5-inch 22-gauge spinal needle is advanced in plane under real-time ultrasound guidance, and 30 mL of 5% dextrose with 0.1% lignocaine is distributed across five predefined target planes.

Cadaveric feasibility was assessed in four lower limbs using 30 mL of 0.1% methylene blue delivered under ultrasound guidance according to the described technique. Gross layered dissection demonstrated dye staining of the intended target structures together with substantial extension into adjacent fascial, periarticular, perineural, and perivascular planes, with proximal interfascial spread along the posterior thigh. Histologic confirmation of intraneural or intravascular spread was not performed. These findings support preliminary anatomical feasibility of regional access and spread, but do not establish selective targeting, procedural safety, histologic precision, or clinical efficacy.

This technical report describes a reproducible ultrasound-guided posteromedial knee injection technique supported by cadaveric dye-distribution findings, detailed sonoanatomic landmarks, and procedural video documentation. The technique appears anatomically plausible and technically feasible as a regional interfascial-periarticular access approach, but further prospective work is needed to determine reliability, safety, mechanism, targeting specificity, and clinical effectiveness.

## Introduction

Knee osteoarthritis (OA) is a common cause of pain, stiffness, and functional limitation. Contemporary understanding increasingly views OA as a disease of the whole joint rather than a disorder confined to articular cartilage, with involvement of multiple articular and periarticular tissues contributing to symptoms and disability [1].

Fascial tissues are increasingly recognized as biologically active connective tissues involved in force transmission, proprioception, and nociception. In particular, fascial mobility along interfascial shear planes may be relevant to musculoskeletal function and pain generation [2]. The concept of interfascial hydrodissection has gained attention as a potential means of restoring normal gliding between fascial layers [3].

The posteromedial knee contains a dense convergence of stabilizing and force-transmitting structures, including the semimembranosus tendon and its expansions, the popliteus, the oblique popliteal ligament (OPL), the posterior capsule, and adjacent fascial layers. Prior anatomical studies have emphasized the complexity of the posterior knee and the close relationship between these structures, while ultrasound studies have shown that several of these components can be visualized dynamically when an appropriate scanning sequence is used [4-7].

In this report, the term “popliteal fascial retinaculum” is used as an operational descriptor for a posteromedial convergence region composed of recognized fascial, capsuloligamentous, and tendon-related structures, rather than as a formally recognized standalone anatomical entity. This terminology is intended to facilitate procedural description and ultrasound-guided targeting of a functionally convergent posteromedial soft-tissue zone [4-8].

The purpose of this technical report is to describe a reproducible ultrasound-guided popliteal fascial retinaculum injection for symptomatic knee OA, present the relevant posteromedial sonoanatomy, and provide a cadaveric dye-distribution study supporting preliminary anatomical feasibility.

## Technical report

Anatomical rationale

For the purposes of this report, we define the "popliteal fascial retinaculum" as the functional and anatomical convergence of posteromedial fascial layers, ligamentous expansions, and tendon insertions that collectively restrain posterior soft-tissue displacement and provide continuity between the posterior knee and thigh compartments [8]. This term is used as an operational and procedural descriptor rather than as established consensus anatomical nomenclature.

The selected target zone lies at a posteromedial convergence point where several stabilizing and force-transmitting structures meet. Under ultrasound, this region can be identified through the relationship among the semimembranosus tendon, popliteus, posterior joint capsule, proximal superficial medial collateral ligament, and posteromedial tibial cortex. Based on prior anatomical descriptions of the posterior knee, this zone may function as a deep interfascial and periarticular access point through which injectate can communicate with multiple adjacent structures and tissue planes [4-7]. Accordingly, the present technique is more appropriately conceptualized as access to a regional posteromedial fascial-periarticular network than as inherently selective injection of a single discrete structure. The five intended targets at this convergence zone are as follows: the semimembranosus tendon insertion on the posteromedial tibia; the popliteus insertion and adjacent tendon-muscle complex; the oblique popliteal ligament located between the semimembranosus and popliteus-related posterior structures; the posterior joint capsule overlying the posterior tibial cortex; and the proximal portion of the superficial medial collateral ligament [4-7].

Neural structures in relative proximity include the tibial nerve, common peroneal nerve, and articular neural contributions to the posterior knee, including the posterior genicular plexus [9]. Fascial continuity from this region extends proximally toward the hamstring-associated fascial layers and distally toward the crural fascia, providing a plausible anatomical basis for broader interfascial spread [2,4,9]. The intended anatomical targets and their principal sonographic landmarks are summarized in Table 1.

**Table 1:** Intended anatomical targets of the posteromedial popliteal fascial retinaculum injection The table summarizes the five target structures, their anatomical relevance, and their characteristic sonographic landmarks using a high-frequency linear transducer (12-15 MHz).

Target structure	Anatomical relevance	Sonographic landmark
Semimembranosus tendon insertion	Posteromedial stabilizer and fascial anchor	Hyperechoic fibrillar tendon at the posteromedial tibial insertion
Popliteus insertion / popliteus tendon-muscle complex	Dynamic posterior stabilizer with oblique course toward the posterior tibia	Obliquely oriented fibrillar tendon insertion or pennate muscle deep to popliteal artery and soft tissues
Oblique popliteal ligament	Posterior capsuloligamentous reinforcement	Band-like structure between semimembranosus- and popliteus-related posterior structures, reinforcing the posterior joint capsule
Posterior joint capsule	Periarticular soft-tissue interface over posterior tibia	Thin layer overlying the posterior tibial cortex and femoral condyles
Proximal superficial medial collateral ligament	Medial periarticular stabilizer	Superficial linear fibrillar band along the medial joint line

Cadaveric dye-distribution study

Cadaveric feasibility was undertaken as a proof-of-concept anatomical study. Four lower limbs were examined, with one limb selected from each donor to avoid within-donor duplication. Each specimen was positioned prone with the knee placed in slight flexion of approximately 15° using a bolster. This position facilitated access to the posterior knee while preserving sonographic visualization of the target structures.

Following surface palpation and ultrasound confirmation of the relevant landmarks, a 5-inch 22-gauge spinal needle was advanced under continuous ultrasound guidance toward the posteromedial convergence zone. A total of 30 mL of 0.1% methylene blue was injected using a hydrodissection technique, with the injectate distributed across the five intended targets using repeated needle redirection under real-time visualization. Approximately 6 mL was directed to each target site (Video 1) [10]. Because 30 mL of methylene blue was delivered with repeated hydrodissection and sequential needle redirection across five intended target planes, the cadaveric findings should be interpreted as demonstrating regional access and injectate spread rather than strict confinement to individual structures.

**Video 1:** Ultrasound-guided injection of methylene blue into cadaveric popliteal fascial planes A 5-inch spinal needle was used to sequentially target all five components of the popliteal fascial retinaculum: the semimembranosus tendon insertion, the oblique popliteal ligament, the posterior joint capsule, the popliteus tendon insertion, and the proximal medial collateral ligament. The video shows the needle advancement, injectate spread, and hydrodissection of tissue planes under continuous real-time ultrasound guidance. A total of 30 mL of methylene blue was used, with approximately 6 mL delivered to each of the five targets. Abbreviations: SemiM, semimembranosus; POP, popliteus; OPL, oblique popliteal ligament; MCL, medial collateral ligament; CPN, common peroneal nerve. Video courtesy of Professor King Hei Stanley Lam

**Figure 1:**
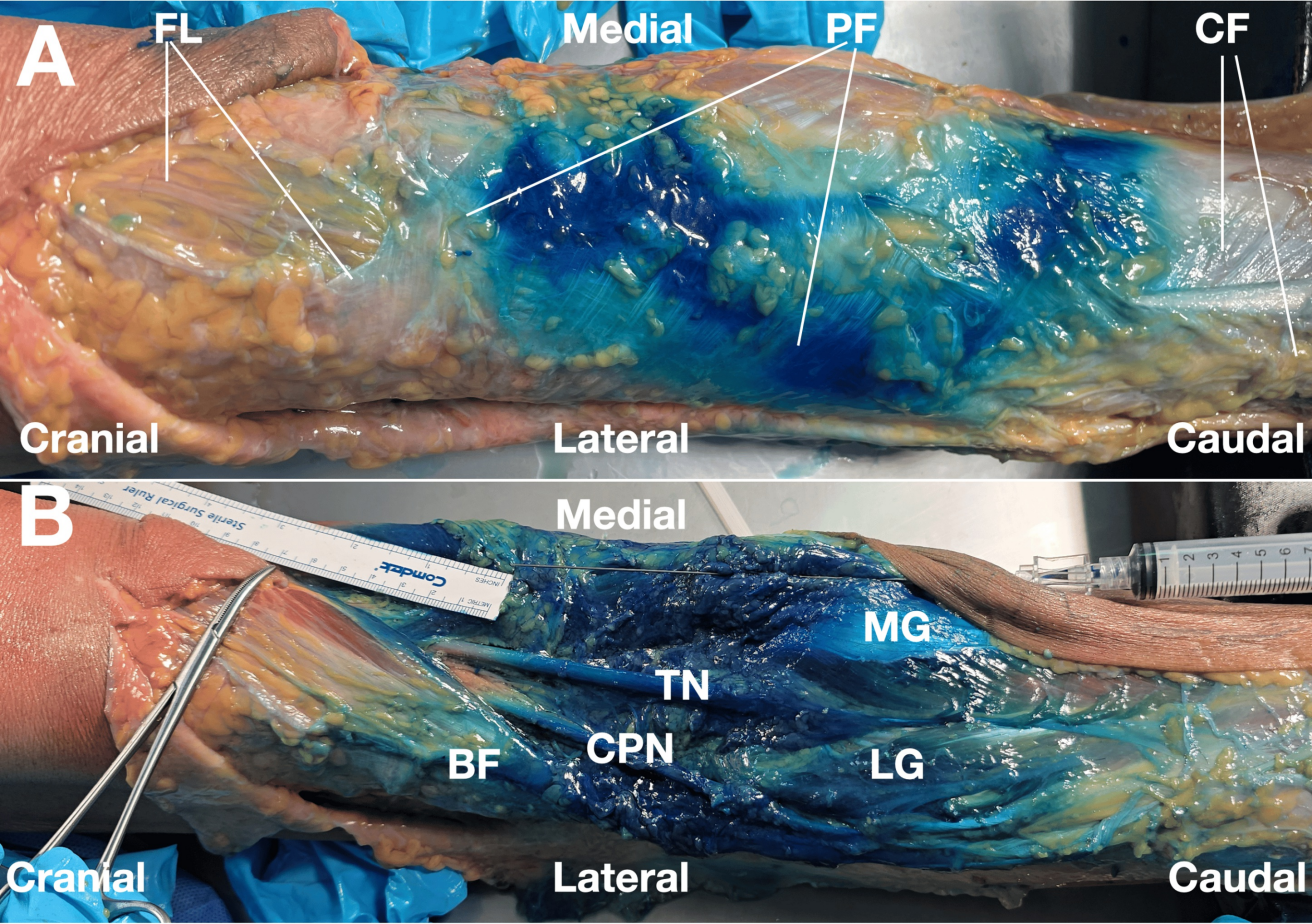
Gross dissection photographs of methylene blue distribution following ultrasound-guided injection targeting the popliteal fascial retinaculum. (A) After removal of the skin and subcutaneous adipose tissue, gross methylene blue distribution to the popliteal fascia is shown before incision of the deeper fascial layers. Dye is visible within the superficial aspects of the popliteal fascial retinaculum. (B) After layered dissection of the popliteal fascia and related structures in the popliteal fossa, methylene blue spread from the medial needle entry site is demonstrated. The total linear spread measured approximately 10 inches (25 cm), extending medially to laterally across the popliteal fascia and toward the biceps femoris/common peroneal nerve region. The five intended target structures are grossly stained, including the popliteus-related insertional region, the oblique popliteal ligament, the semimembranosus insertion, the posterior joint capsule, and the proximal superficial medial collateral ligament. The image also demonstrates that dye spread is not confined to a single focal structure but extends across a broader regional fascial plane. Abbreviations: BF, biceps femoris; CPN, common peroneal nerve; TN, tibial nerve; CF, crural fascia; FL, fascia lata; PF, popliteal fascia; MG, medial head of gastrocnemius; LG, lateral head of gastrocnemius. Image courtesy of Professor King Hei Stanley Lam.

**Figure 2:**
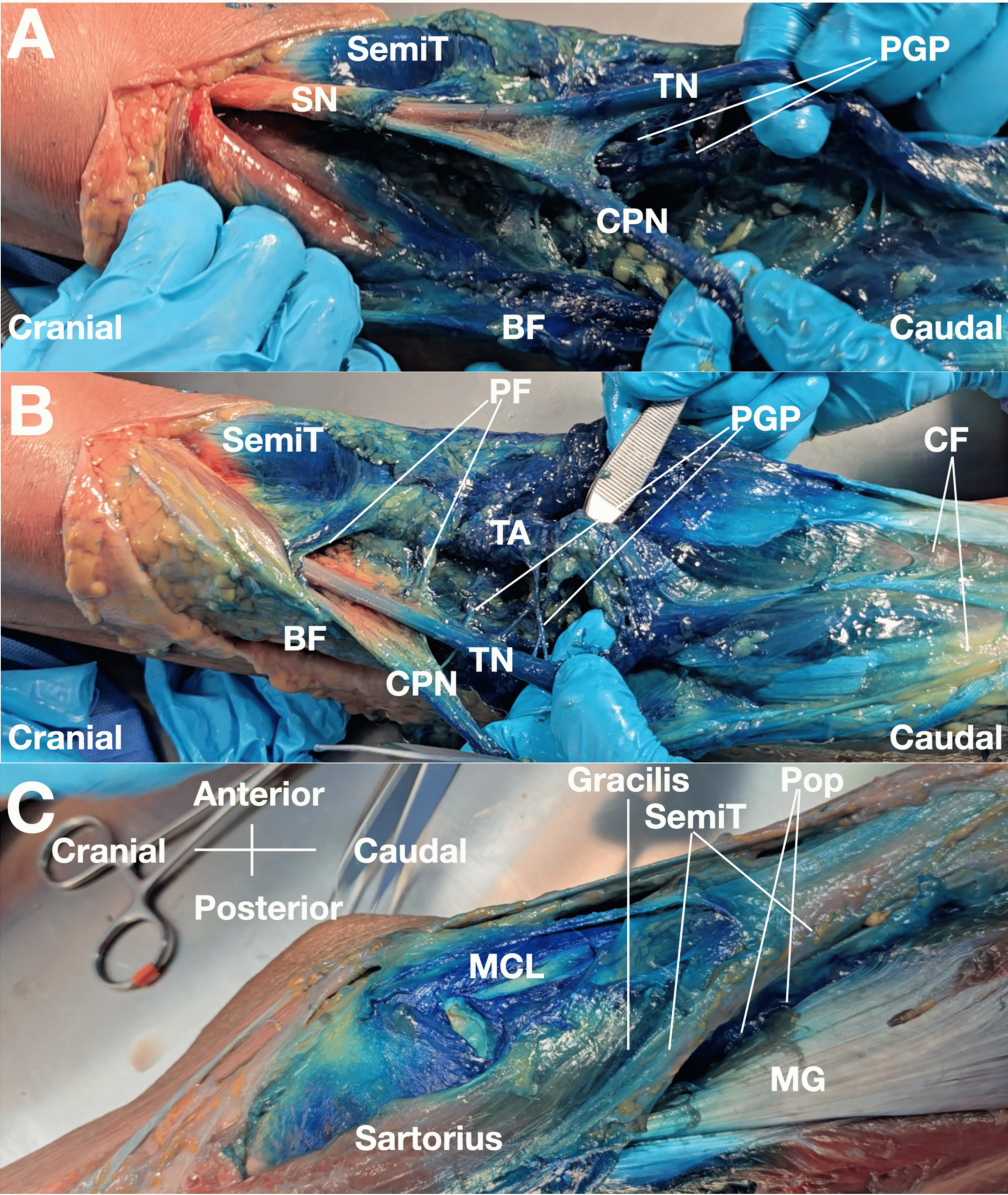
Gross dissection photographs demonstrating methylene blue spread to neural and vascular structures following ultrasound-guided injection targeting the popliteal fascial retinaculum (A) Methylene blue grossly stains the region of the sciatic bifurcation and surrounds the tibial nerve and common peroneal nerve. Dye tracks along the external neural planes; however, histologic assessment was not performed, and intraneural exclusion cannot be confirmed. Gross staining of epineural/perineural surfaces does not confirm intraneural safety and should be interpreted as a potential safety concern rather than as evidence of therapeutic neural targeting. (B) Methylene blue extends to the region of the posterior genicular plexus and surrounds the tibial artery, demonstrating perivascular and perineural spread in the posterior knee. (C) Methylene blue stains the medial collateral ligament and posterior joint capsule, demonstrating dye presence within periarticular soft-tissue targets [4,9]. Abbreviations: BF, biceps femoris; CPN, common peroneal nerve; PGP, posterior genicular plexus; SemiT, semitendinosus; SN, sciatic nerve; PF, popliteal fascia; CF, crural fascia; TN, tibial nerve; TA, tibial artery; Pop, popliteus; MG, medial head of gastrocnemius; MCL, medial collateral ligament. Image courtesy of Professor King Hei Stanley Lam.

Immediate layered dissection was subsequently performed by an experienced anatomist to document gross dye distribution within the subcutaneous tissue, popliteal fascial layers, periarticular structures, and adjacent tissue planes (Figures 1, 2).

Ultrasound equipment and landmark identification

A high-frequency linear transducer in the 12-15 MHz range was used throughout the procedure. Sonographic assessment began in the transverse plane to identify the popliteal vessels and tibial nerve as critical avoidance structures. Scanning then focused on the posteromedial knee to localize the semimembranosus insertion, popliteal fascial planes, OPL, posterior capsule, proximal superficial medial collateral ligament, and adjacent posteromedial tibial cortex [5-7].

Once these structures had been identified, the transducer was rotated into a longitudinal orientation to optimize visualization of the target convergence zone while maintaining a working corridor away from the central popliteal neurovascular bundle [6,9].

Step-by-step injection technique

The procedure is performed under sterile conditions. In clinical use, each patient receives three monthly treatment sessions.

Step 1: Patient Positioning

The patient is placed prone with a bolster under the ankle to maintain knee flexion at approximately 15°. This reduces soft-tissue crowding in the popliteal fossa and improves access to the posteromedial knee.

Step 2: Transverse-Plane Landmark Identification

**Figure 3:**
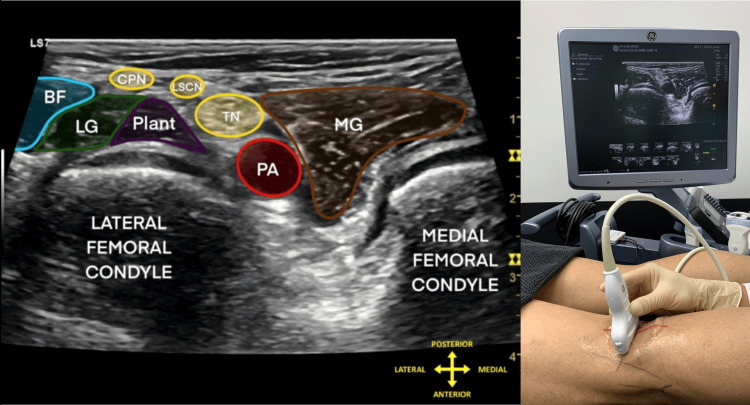
Transverse safety scan of the popliteal fossa showing the neurovascular bundle and structures to avoid Representative transverse ultrasound image of the popliteal fossa demonstrating the key safety landmarks that should be identified before needle advancement. The popliteal artery, popliteal vein, and tibial nerve are visualized first as critical avoidance structures. Depending on scan level, the common peroneal nerve and lateral sural cutaneous nerve may also be identified. This initial safety scan establishes orientation and helps define a safer posteromedial working corridor for subsequent target localization and injection. Abbreviations: BF, biceps femoris; CPN, common peroneal nerve; LSCN, lateral sural cutaneous nerve; TN, tibial nerve; PA, popliteal artery; LG, lateral head of gastrocnemius; MG, medial head of gastrocnemius. Image courtesy of Professor King Hei Stanley Lam and adapted from NYSORA educational material with permission [11].

**Figure 4:**
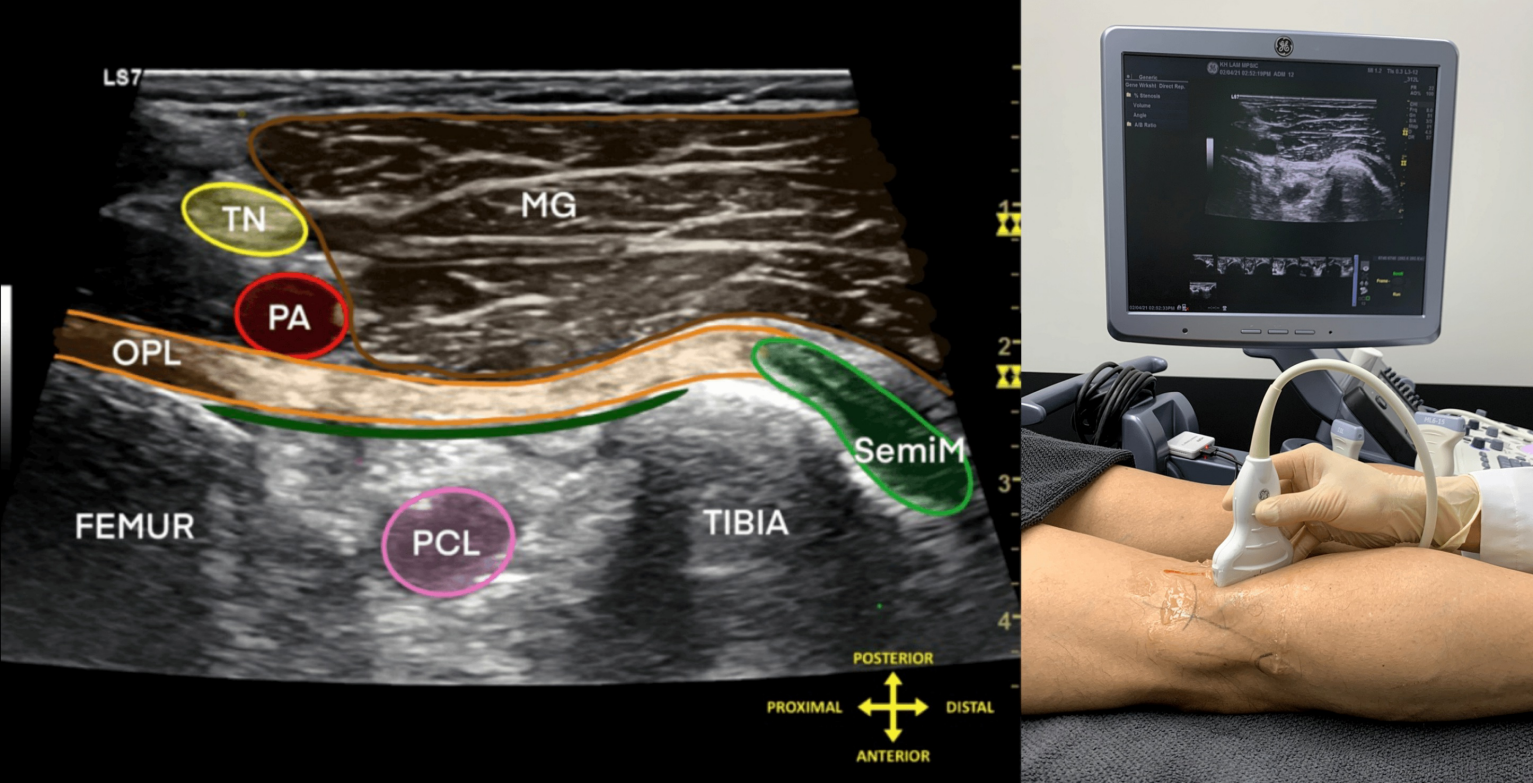
Oblique transverse ultrasound scan demonstrating the oblique popliteal ligament Oblique transverse ultrasound image obtained over the posteromedial knee demonstrating the oblique popliteal ligament within the posterior capsuloligamentous complex. This view serves as an intermediate orientation step between the initial transverse safety scan and the more target-specific longitudinal view. The corresponding probe position and hand position are shown to facilitate reproducibility. Abbreviations: OPL, oblique popliteal ligament; TN, tibial nerve; PA, popliteal artery; MG, medial head of gastrocnemius; PCL, posterior cruciate ligament; SemiM, semimembranosus. Image courtesy of Professor King Hei Stanley Lam and adapted from NYSORA educational material with permission [11].

With the transducer in the transverse plane over the popliteal fossa, the popliteal vessels and tibial nerve are identified first as critical avoidance structures (Figure 3). Scanning is then directed posteromedially to identify the semimembranosus tendon, popliteal fascial layers, OPL, posterior capsule, proximal superficial medial collateral ligament, and the posteromedial tibial cortex. An oblique transverse scan is then used to better define the OPL (Figure 4) [6,7].

Step 3: Longitudinal Target Localization

**Figure 5:**
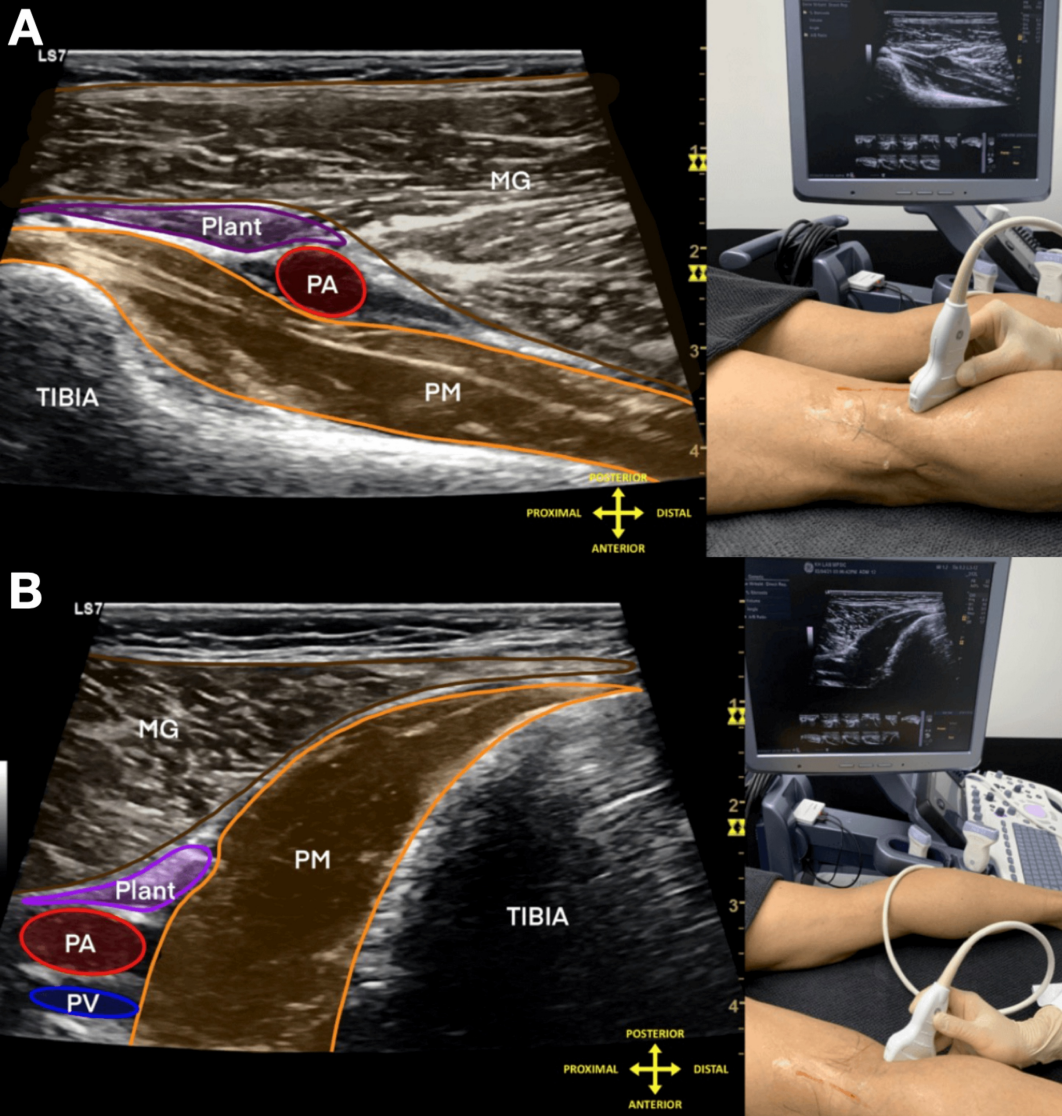
Oblique transverse long-axis ultrasound views of the popliteus muscle from the mid portion to its tibial insertion (A) Oblique transverse long-axis ultrasound image demonstrating the mid portion of the popliteus muscle. (B) More distal oblique transverse long-axis ultrasound image demonstrating the insertion of the popliteus muscle at the posterior tibia. These paired images illustrate the sonographic course of the popliteus from its muscular mid portion to its tibial insertion and help define its relationship to adjacent posteromedial knee structures. The corresponding probe position and hand position are shown to facilitate reproducibility of the scanning technique. Abbreviations: MG, medial head of gastrocnemius; Plant, plantaris; PA, popliteal artery; PM, popliteus muscle; PV, popliteal vein. Image courtesy of Professor King Hei Stanley Lam and adapted from NYSORA educational material with permission [11].

**Figure 6:**
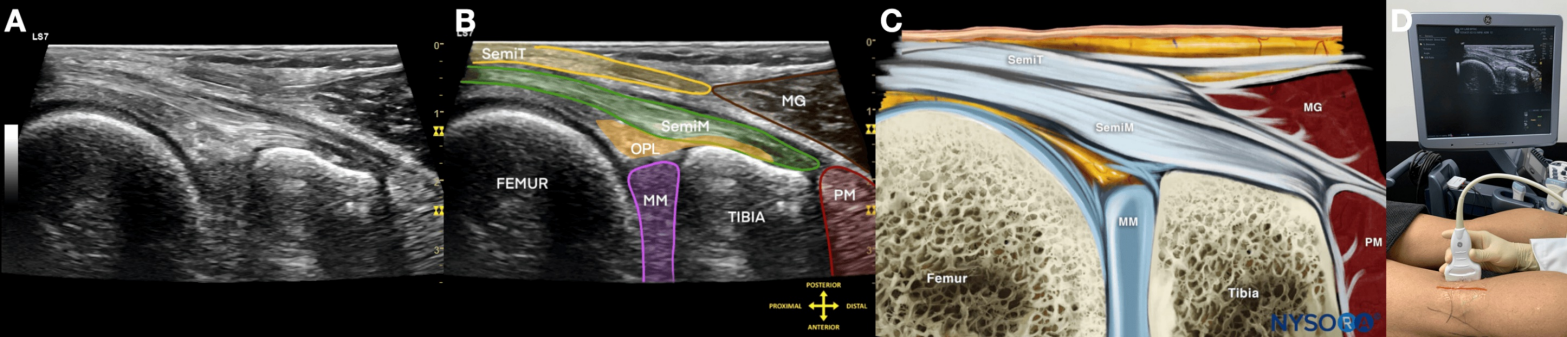
Longitudinal ultrasound localization of the posteromedial convergence zone at the semimembranosus and popliteus insertions Composite figure demonstrating the key longitudinal target view used for final procedural localization. (A) B-mode ultrasound image showing the convergence region over the insertion of the semimembranosus and popliteus. (B) Color-shaded version of the same image highlighting the corresponding sonoanatomy. (C) Schematic drawing of the corresponding anatomical structures. (D) External view showing the relevant probe position, operator hand position, patient position, and surface landmarks used to obtain this view. This longitudinal convergence-zone image is the principal target-localization view for the procedure. Abbreviations: MG, medial head of gastrocnemius; PM, popliteus muscle; SemiT, semitendinosus; SemiM, semimembranosus; MM, medial meniscus. Image courtesy of Professor King Hei Stanley Lam and adapted from NYSORA educational material with permission [11].

The popliteus is first traced in an oblique transverse long-axis view from its mid portion toward its tibial insertion (Figure 5A-5B). The transducer is then rotated into a longitudinal or sagittal plane to visualize the convergence of the semimembranosus, OPL-related posterior structures, and popliteus-related insertional region over the posterior tibia (Figure 6) [4-7].

Step 4: Needle Advancement and Hydrodissection

**Figure 7:**
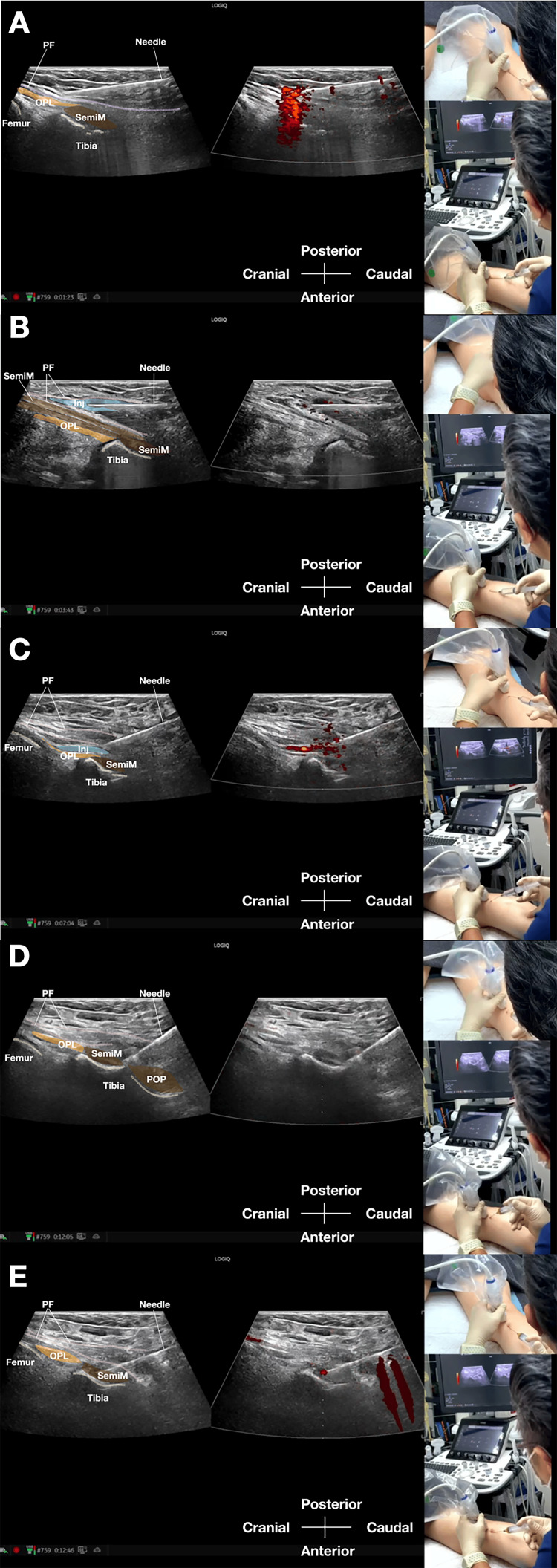
Real-patient ultrasound-guided popliteal fascial retinaculum injection demonstrating sequential target planes Representative real-patient ultrasound-guided procedural still images demonstrating in-plane needle placement and injectate delivery at the principal target planes of the popliteal fascial retinaculum injection. (A) Injection targeting the superficial popliteal fascia. (B) Injection targeting the plane between the superficial and middle layers of the popliteal fascia. (C) Injection targeting the oblique popliteal ligament. (D) Injection targeting the junction between the semimembranosus and popliteus insertions. (E) Injection targeting the insertion of the semimembranosus tendon. Panels A-E are still frames extracted from Video 2 and presented with color shading and labels for educational clarification of target anatomy and needle-tip position. These images are presented for procedural illustration only and should not be interpreted as evidence of clinical efficacy or safety. Abbreviations: Inj, injectate; MG, medial head of gastrocnemius; OPL, oblique popliteal ligament; POP, popliteus; PF, popliteal fascia; SemiT, semitendinosus; SemiM, semimembranosus. Image courtesy of Professor King Hei Stanley Lam.

Using a strict in-plane distal-to-proximal approach, a 5-inch 22-gauge spinal needle is advanced under continuous real-time ultrasound guidance toward the target junction. In leaner patients, a 3.5-inch needle may be adequate; the 5-inch needle is preferred when deeper soft-tissue penetration is required. After negative aspiration confirms extravascular placement, 30 mL of 5% dextrose with 0.1% lignocaine is injected. The injectate is divided approximately equally among the five targets, with about 6 mL delivered to each site while the needle tip is dynamically repositioned under continuous ultrasound visualization. Hydrodissection is used to separate tissue planes and facilitate distribution across the intended interfascial and periarticular structures. The 30 mL volume was intentionally selected to permit broad hydrodissection across multiple contiguous posteromedial tissue planes; however, this volume likely also contributes to non-selective spread beyond the intended structures and therefore limits conclusions regarding target specificity. Five percent dextrose with 0.1% lignocaine was selected for its primary role as a hydrodissection medium combined with local anesthetic effect rather than as a sclerosant prolotherapy agent [10,11]. Representative real-patient still images are shown in Figure 7A-7E, and the complete dynamic procedural sequence is provided in Video 2 [10].

**Video 2:** Real-patient ultrasound-guided popliteal fascial retinaculum injection demonstrating sequential target-plane injectate delivery Real-time ultrasound-guided procedural video demonstrating the popliteal fascial retinaculum injection in a patient using an in-plane approach. The video shows dynamic needle advancement and sequential injectate delivery to the superficial popliteal fascia, the plane between the superficial and middle popliteal fascial layers, the oblique popliteal ligament, the junction between the semimembranosus and popliteus insertions, and the semimembranosus insertion. Annotated still frames extracted from this video are presented in Figure 7A-7E to highlight the principal target planes and needle-tip locations. This video is included for technical demonstration only and does not constitute evidence of clinical effectiveness or safety. Abbreviations: Inj, injectate; MG, medial head of gastrocnemius; OPL, oblique popliteal ligament; POP, popliteus; PF, popliteal fascia, SemiT, semitendinosus; SemiM, semimembranosus. Image and video courtesy of Professor King Hei Stanley Lam.

Step 5: Post-procedure Care

The needle is withdrawn, light pressure is applied, and a sterile dressing is placed. Patients are advised to avoid strenuous activity for 48 hours while continuing gentle, pain-free range-of-motion exercises. Patients should also be advised not to drive or operate machinery for at least 60 minutes after the procedure. This sequence is repeated monthly for a total of three treatment sessions. The procedural workflow is summarized in Table 2.

**Table 2:** Step-by-step procedural workflow for ultrasound-guided popliteal fascial retinaculum injection The table outlines eight sequential steps from patient positioning to post-procedure care, with the practical purpose of each maneuver. The procedure is performed under continuous real-time ultrasound guidance using a 5-inch 22-gauge needle and 30 mL of 5% dextrose with 0.1% lignocaine delivered via hydrodissection. In clinical use, three monthly treatment sessions are administered.

Step	Maneuver	Practical purpose
1	Prone positioning with knee flexed approximately 15°	Improves access and reduces popliteal fossa soft-tissue tension
2	Transverse scan to identify popliteal vessels and tibial nerve	Establishes safety landmarks; optional skin marking of the projected vascular course may be used to reinforce safety orientation
3	Posteromedial landmark identification	Defines target structures and the intended needle trajectory
4	Rotation to longitudinal plane	Optimizes visualization of the convergence zone
5	In-plane distal-to-proximal needle advancement	Maintains continuous visualization of shaft and tip
6	Hydrodissection with 30 mL of 5% dextrose with 0.1% lignocaine	Separates tissue planes and distributes injectate
7	Dynamic redirection across five targets	Improves coverage of intended periarticular structures
8	Post-procedure dressing and activity modification	Routine aftercare; advise no driving for 60 minutes

Technical pearls

Several practical observations emerged during development of this technique. First, slight knee flexion improved sonographic access to the posterior knee and reduced crowding within the popliteal fossa. Second, early identification of the popliteal vessels and tibial nerve in the transverse plane provided an essential safety reference before needle advancement. Third, hydrodissection served not only as a means of fluid delivery but also as a real-time indicator of tissue-plane separation. Finally, controlled needle redirection was preferable to forceful single-point injection because it allowed more even distribution across the intended periarticular targets [6,10].

Safety considerations

Continuous real-time ultrasound guidance is mandatory to reduce the risk of injury to the popliteal vessels and tibial nerve. Aspiration should be performed before each injection to help confirm that the needle tip is not intravascular. A 5-inch needle may be useful for reaching deeper targets in patients with a larger body habitus, and the injectate should be delivered slowly with continuous monitoring of needle-tip position and patient tolerance.

Potential procedural risks include vascular injury, neural irritation, bleeding or hematoma formation, infection, and transient post-injection pain. Although the planned needle trajectory is intended to remain separate from the main popliteal neurovascular bundle, cadaveric dye spread in this study extended into adjacent perineural and perivascular planes. Histologic confirmation was not performed. Therefore, gross neural- or vascular-adjacent staining cannot distinguish perineural from intraneural spread, or perivascular from intravascular spread, and should not be interpreted as evidence of safety. Accordingly, this report should not be interpreted as a formal safety study, and the clinical implications of such spread remain uncertain [9].

Cadaveric findings

Layered dissection demonstrated gross staining of all intended target structures. Methylene blue was identified at the semimembranosus insertion, popliteus-related insertional region, posterior capsule, OPL, and proximal superficial medial collateral ligament. Dye was also visible within the popliteal fascial layers, supporting communication between the targeted periarticular region and the multilayered fascial system of the posterior knee (Figures 1, 2) [4,7]. However, the observed distribution was regional rather than strictly target-confined.

Gross dye spread was not limited to the immediate target structures. Dye was observed adjacent to the bifurcation of the sciatic nerve and around the tibial and common peroneal nerves. Extension of dye was also observed in the region of the posterior genicular plexus and around the tibial artery. In addition, methylene blue stained the medial collateral ligament and posterior joint capsule [8]. These findings indicate broad interfascial and periarticular communication but also reduce confidence in target specificity and raise safety questions that cannot be resolved by gross dissection alone.

Proximal extension of methylene blue was observed along interfascial planes associated with the posterior thigh musculature, including the hamstring-related fascial compartments. This proximal spread measured approximately 25 cm from the needle entry point. Taken together, these findings suggest that the posteromedial access point may permit entry into a broader interfascial and periarticular network rather than remaining confined to a single insertional structure [2,4]. This wide extent of spread may reflect the combined effects of the access location, repeated hydrodissection, injectate volume, and cadaveric tissue characteristics.

Importantly, when the described sonographic landmarks and in-plane approach were respected, the planned needle trajectory remained separate from the popliteal vessels and tibial nerve. However, because histologic analysis was not performed, we cannot rule out microscopic intraneural or intravascular injectate spread, even when gross dissection suggested perineural or perivascular distribution. Accordingly, these observations should be interpreted as anatomical feasibility findings rather than proof of procedural safety. The gross cadaveric findings are summarized in Table 3.

**Table 3:** Summary of gross cadaveric dye-distribution findings Summary of gross cadaveric dye-distribution findings following ultrasound-guided injection of 30 mL of 0.1% methylene blue targeting the popliteal fascial retinaculum in four lower limbs. Dye was identified at all five intended target structures, within popliteal fascial layers, adjacent to perineural and perivascular structures (sciatic nerve bifurcation, tibial nerve, common peroneal nerve, posterior genicular plexus, and tibial artery), at the medial collateral ligament and posterior joint capsule, and along proximal interfascial planes extending approximately 25 cm. The planned needle trajectory remained separate from the main popliteal vessels and tibial nerve. Histologic confirmation was not performed. These findings support anatomical plausibility but not therapeutic efficacy or clinical safety.

Observation	Gross finding
Intended target staining	Dye identified at all five intended target structures
Fascial layer involvement	Dye visible within popliteal fascial layers
Perineural/perivascular spread	Dye observed adjacent to the sciatic bifurcation, tibial nerve, common peroneal nerve, posterior genicular plexus, and tibial artery
Periarticular spread	Dye present at the medial collateral ligament and posterior joint capsule
Proximal extension	Dye tracked proximally along posterior thigh interfascial planes for approximately 25 cm
Needle trajectory safety	Planned needle path remained separate from the main popliteal vessels and tibial nerve when described landmarks were followed

Brief clinical experience note

This technique was subsequently applied in a separate retrospective case series of patients with Kellgren-Lawrence grade 1-3 knee osteoarthritis, each receiving three monthly ultrasound-guided 5% dextrose with 0.1% lignocaine injections using the described approach. Clinical outcome evaluation is outside the scope of the present technical report and is being addressed separately.

## Discussion

This report describes a novel ultrasound-guided posteromedial knee injection technique supported by a cadaveric dye-distribution study, sequential landmarking images, and procedural video documentation. Its principal contribution is technical rather than inferential: it defines a reproducible scanning sequence, emphasizes early safety mapping of the popliteal neurovascular bundle, and demonstrates a layered injectate-distribution strategy across a posteromedial periarticular and interfascial convergence zone. The report should therefore be interpreted as a technical and anatomical feasibility study rather than as evidence of selective targeting, clinical safety, or therapeutic efficacy.

A particular strength of the report is the integration of static ultrasound teaching images with two complementary videos. Video 1 demonstrates methylene-blue injection in cadaveric popliteal fascial planes, while Video 2 demonstrates real-patient ultrasound-guided targeting of the principal injection planes. Together with Figures 1-7, these media are intended to improve technical clarity and reproducibility.

The cadaveric findings support anatomical plausibility but require cautious interpretation. Dye spread in cadaveric tissue does not establish pharmacologic effect, tissue specificity, or clinical safety. Neural- and vascular-adjacent staining should be considered a potential safety concern rather than a therapeutic advantage. Likewise, gross staining near neural or vascular structures does not constitute evidence of therapeutically meaningful hydrodissection or atraumatic perineural distribution. Histologic confirmation was not performed.

An important interpretive issue is targeting specificity. The dye-distribution findings indicate that the injectate was not confined to the five intended structures but instead spread across adjacent interfascial, periarticular, perineural, and perivascular planes. This pattern likely reflects the combined influence of the posteromedial access point, dynamic needle redirection, hydrodissection, and the relatively large injectate volume. The technique may therefore be more accurately viewed as an approach to a regional posteromedial fascial-periarticular network than as a strictly structure-specific injection.

The terminology used for the injectate also warrants caution. According to current dextrose-injection literature, 5% dextrose is more commonly associated with perineural injection therapy and hydrodissection than with classical hypertonic prolotherapy, which more typically uses higher concentrations [10,12]. The addition of dilute lignocaine was intended to improve procedural comfort; however, the specific contribution of this adjunct was not evaluated in the present study. Any therapeutic mechanism, if present, remains hypothetical and may relate to mechanical hydrodissection, temporary alteration of interfascial gliding, regional fluid redistribution, indirect perineural exposure, or other non-specific local effects. The present cadaveric observations do not permit mechanistic discrimination among these possibilities. For this reason, the present manuscript is framed as a technical report describing an ultrasound-guided interfascial and periarticular procedure rather than a definitive mechanistic or efficacy study.

Future work should include prospective reliability studies, formal safety assessment, quantitative spread analysis, and controlled clinical trials.

Limitations

This report has several limitations. First, the cadaveric component involved only four lower limbs. Second, the dissection findings were gross rather than histologic. Third, because histologic analysis was not performed, we cannot rule out microscopic intraneural or intravascular injectate spread. Fourth, cadaveric dye spread may not accurately reflect injectate behavior in living tissue. Fifth, the study did not include quantitative mapping or standardized compartment-based analysis of dye distribution, limiting precision and reproducibility of interpretation. Sixth, the relatively large injectate volume and use of hydrodissection likely contributed to broad regional spread thereby limiting conclusions regarding targeting specificity. Seventh, the manuscript does not establish clinical safety or efficacy. Eighth, no in vivo validation or formal safety study was performed. Ninth, the brief clinical note is intentionally limited to avoid overlap with separate clinical outcome reporting.

## Conclusions

This technical report describes a reproducible ultrasound-guided popliteal fascial retinaculum injection technique for the posteromedial knee, supported by cadaveric dye-distribution findings and structured sonoanatomic landmarks. The technique appears anatomically feasible and practically teachable when performed with careful attention to posterior knee safety anatomy and continuous real-time ultrasound guidance. However, the cadaveric findings also demonstrate broad regional spread beyond the intended targets, including neural- and vascular-adjacent planes. Accordingly, the present report should not be interpreted as establishing selective targeting, procedural safety, mechanism, or clinical effectiveness. Further study is required to determine reliability, safety, mechanism, quantitative spread characteristics, and clinical effectiveness.
